# Ion Microscope Imaging Mass Spectrometry Using a Timepix3-Based Optical Camera

**DOI:** 10.1021/jasms.2c00223

**Published:** 2022-11-16

**Authors:** Daniel Wood, Robert J. Burleigh, Natasha Smith, Daniela Bortoletto, Mark Brouard, Michael Burt, Andrei Nomerotski, Richard Plackett, Ian Shipsey

**Affiliations:** † Robert Hooke Building, Department of Physics, University of Oxford, Parks Road, OxfordOX1 3PP, United Kingdom; ‡ Chemistry Research Laboratory, Department of Chemistry, University of Oxford, 12 Mansfield Road, OxfordOX1 3TA, United Kingdom; ¶ Brookhaven National Laboratory, Upton, New York11973, United States

**Keywords:** mass spectrometry imaging, stigmatic ion imaging, ion microscope imaging, fast imaging sensor

## Abstract

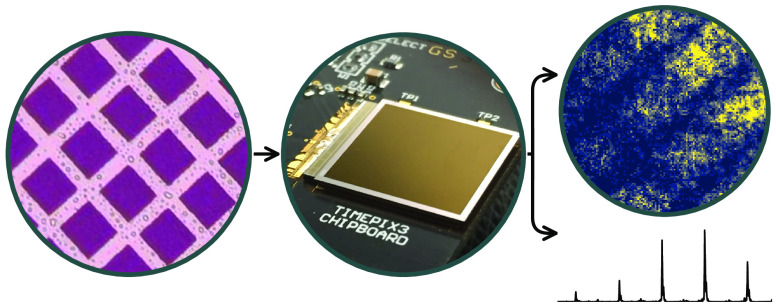

Ion microscopy allows for high-throughput mass spectrometry imaging. In order to resolve congested mass spectra, a high degree of timing precision is required from the microscope detector. In this paper we present an ion microscope mass spectrometer that uses a Timepix3 hybrid pixel readout with an optimal 1.56 ns resolution. A novel triggering technique is also employed to remove the need for an external time-to-digital converter (TDC) and allow the experiment to be performed using a low-cost and commercially available readout system. Results obtained from samples of rhodamine B demonstrate the application of multimass imaging sensors for microscope mass spectrometry imaging with high mass resolution.

## Introduction

Surface imaging is a key element of many analytical methods. Ion microscopy is distinctly beneficial for high-throughput mass spectrometry imaging (MSI), where a defocused ionizing beam is used to simultaneously analyze relatively large areas, around 0.05 to 100 mm^2^.^1−6^ Ions generated in this way are extracted from the sample surface using a strong electric field to preserve their initial spatial information and imaged onto a position-sensitive detector by the ion microscope. The ions are separated by their times-of-flight and sequentially arrive at the detector, producing ion images of the individual surface components.

Multiturn or reflectron ion microscopes can reach mass resolutions of 10^5^–10^6^*m*/Δ*m*.^4,5,7−10^ An ideal detector should therefore measure location-specific information with subnanosecond timing, while avoiding saturation due to high count rates. These criteria are challenging but are broadly met by combining microchannel plates (MCPs) with time-stamping complementary metal oxide semiconductor (CMOS) arrays.^11,12^ Ions impacting MCPs generate a cascade of electrons, which can be directly detected by a CMOS sensor placed in vacuum or converted to light by a scintillator and counted externally.

Time-stamping sensors pair well with the event-driven nature of MSI, as detected ions can be integrated over their times-of-flight to produce stigmatic images or over their positions to analyze mass spectra from specific locations, as in a sample well assay.^10,11,13−18^ They improve on purely position-sensitive sensors by allowing for multimass imaging during a single experimental cycle. Several generations of Pixel Imaging Mass Spectrometry (PImMS) and Medipix/Timepix CMOS sensors have applied these techniques with timing precisions down to 10 ns.^19−22^ However, ion microscope time-of-flight spectra are typically on the order of 10–100 μs, which limits the effective mass resolution of the sensors to ∼10^3^*m*/Δ*m*.

This report presents the results of using the Timepix3 hybrid pixel readout chip,^23^ which has an ultimate time precision of 1.5625 ns, to perform laser desorption MSI with a reflectron ion microscope.^10^ This combination represents a significant potential improvement in attainable mass resolution compared to previous multimass spatial imaging experiments. Internal trigger markers corresponding to the arrival times of the laser pulses were additionally used to remove the need for an external time-to-digital converter (TDC), which allowed these experiments to be performed using a low-cost, commercially available readout system, the Advacam AdvaDAQ.^24^ This approach enabled time-of-flight differences to be calculated relative to an equivalent photon energy distribution, which reduced the effect of timewalk and increased the achievable mass resolution.

## Methods

### Timepix3 Camera

The Timepix3 hybrid pixel readout chip was developed by the Medipix3 collaboration.^20,23^ It comprises a 256 × 256 array of square pixels with 55 μm pitch. Each pixel has the ability to record simultaneous time-of-arrival (ToA) and time-overthreshold (ToT) data when triggered. The 10-bit ToT and 18-bit ToA data are recorded by a 40 MHz global reference clock distributed across the entire pixel matrix. This provides ToT and coarse ToA with a resolution of 25 ns. Four additional bits of fine ToA data are provided by local voltage-controlled oscillators running at 640 MHz, refining the resolution to 1.56 ns. Threshold dispersion is minimized across the chip by a 4-bit digital-to-analogue converter within each pixel, which allows for local threshold adjustment.

The Timepix3 chip can be operated in one of two modes. The first is a standard sequential frame mode where all pixels are read at the end of the shutter period. The second is a data-driven mode in which the chip outputs a 48-bit packet each time a pixel records an event. This zero-suppression optimizes bandwidth, allowing for a maximum rate of 40 Mhits s^–1^ cm^–2^. In this work, data-driven pixel readout was provided by the AdvaDAQ USB 3.0 readout system for Timepix3 (Advacam),^24^ with control and data acquisition handled by the accompanying PiXet software.

Timepix3 chips are typically bump-bonded to standard silicon sensors for use as X-ray detectors or particle trackers. However, this work required detection of optical photons from a scintillator with an emission maximum at 423 nm.^25^ Therefore, a sensor containing a thinned entrance window and an antireflective coating was used.^26^ This provides high quantum efficiency over the range 400–900 nm.^27^ The camera used a 55 mm f/2.8 macro lens (Nikon) to focus light from the scintillator onto the sensor.

### Mass Spectrometer

The Timepix3 camera was coupled to a purpose-built time-of-flight microscope imaging mass spectrometer. The instrument is a two-stage system where the ion microscope creates an image plane that serves as a pseudosource for a gridless reflectron.^10^ The postextraction differential acceleration technique was used to temporally focus this image plane for a particular *m*/*z* without compromising the attainable spatial resolution.^28^ Under the 22 V mm^–1^ extraction field employed here, ion images have been recorded with mass and spatial resolutions of 8100 ± 700 *m*/Δ*m* and 14 μm over 300–600 Da using a gated CCD camera.^10^

Rhodamine B samples were electrosprayed onto conductive 25 mm × 25 mm indium tin oxide surfaces. The samples were covered with a nickel mesh during the coating process to produce grid patterns with 40 μm gaps and 63 μm pitches. Each sample was individually placed into a slot in the repeller electrode of the ion microscope and ablated by 355 nm light incident at ∼20° from a 10 Hz Nd:YAG laser (Continuum Powerlight 8010). The object plane of the generated ions was then electrostatically projected through the ion microscope and reflectron and onto a detector comprising dual-stack MCPs and an Exalite 404 scintillator. This produced flashes of light that were recorded outside of vacuum by Timepix3. The emission lifetime of the scintillator limits the instrument response time to approximately 3.7 ns.^10,25^

### Trigger Marking

**Figure 1:**
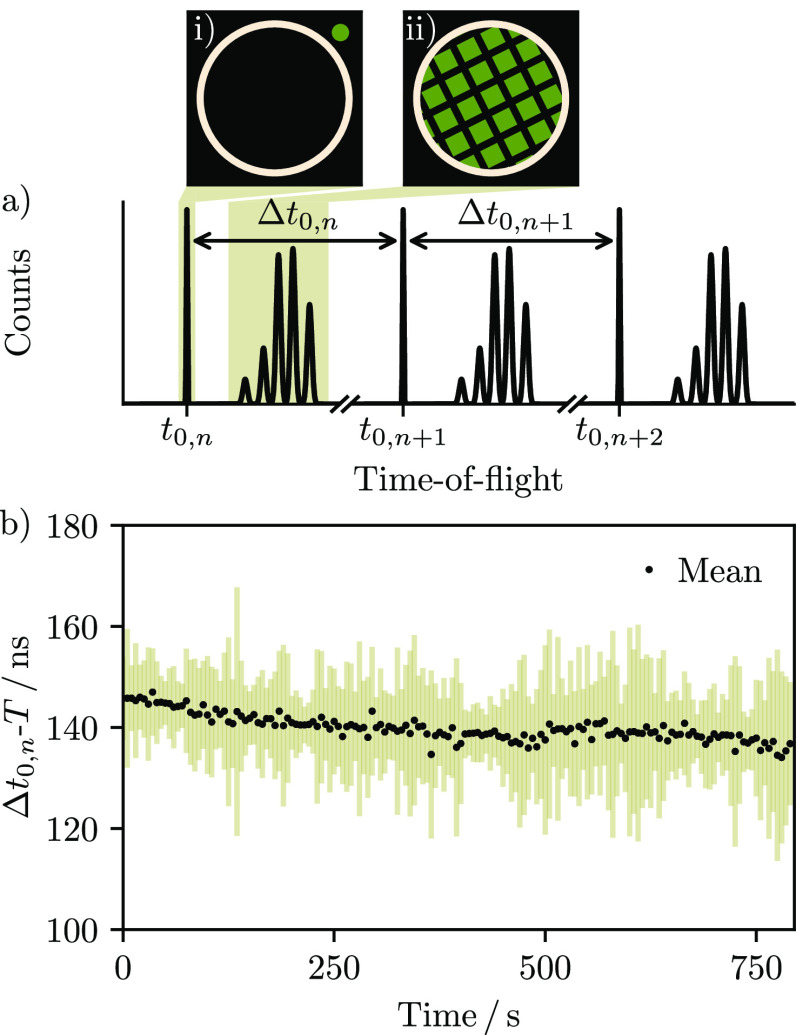
(a) The data-driven mode of Timepix3 provides output each time a pixel is activated, in this case producing a series of repeating time-of-flight spectra at 10 Hz. Integrating over ToA (shaded regions) allows images of the laser light (i) and grid-patterned ion signal (ii) to be distinguished. The spectra are averaged without the need for an external trigger by referencing each to an internal zero (*t*_0,*n*_) corresponding to the arrival of the *n*th laser shot. The laser beam is split and focused onto an area of the sensor (green circle, (i)) outside of the region occupied by light from the scintillator (tan circle) to allow it to be automatically identified for each laser shot. This is necessary because the time between each reference point (*Δt*_0,*n*_) varies slightly during the experiment. (b) Subtracting *Δt*_0,*n*_ by the nominal pulse period (*T* = 100 ms) provides information on the shot-to-shot stability. For example, binning data into 5 s groups (i.e., 50 laser shots) demonstrates that the average standard deviation (tan lines) is approximately 16 ns and that the laser runs 1.4 × 10^–4^% slower than 10 Hz (black circles).

The nominal timing resolution of Timepix3 becomes difficult to achieve when measuring relative to an external trigger, because the state of the global 40 MHz reference clock is unknown and results in an inherent jitter in the acknowledgment of the trigger signal by the chip. This led to the development of readout systems incorporating external TDCs, which provide additional time stamping of each event.^29,30^ As such systems are costly and relatively complex, in this work we instead make use of a portion of the laser pulse itself to define initiation times *t*_0,*n*_ for each of the *n* measured time-of-flight spectra. A beam splitter was placed in the path of the laser beam, which directed a small part of the light through an optical fiber and onto a well-defined region of the sensor, as shown schematically in Figure 1a. This provided a spatially stable trigger marker at the beginning of each time-of-flight spectrum from which the *t*_0,*n*_ could be determined.

As the period *T* between laser pulses (100 ms) is much longer than the measured time-of-flight spectra (∼20 μs), the data-driven output can be split into individual frames corresponding to each of the *n* laser shots. This was accomplished using K-means sorting,^31,32^ which allowed each time-of-flight spectrum to be referenced to the appropriate *t*_0_. An average time-of-flight spectrum was then produced by summing over all laser shots. The temporal stability of the optical pulse is shown in Figure 1b. Averaging over groups of 50 laser shots demonstrates that the range on the time interval between each recorded trigger marker is 100.000140 ± 0.000016 ms. This data can in principle also be used to filter output by pulse arrival time.

An internal *t*_0_ of this kind is also advantageous in that it reduces the effect of timewalk on ToA measurements when the overall ToT distribution is narrow, as in our case. Timewalk arises when varying amounts of charge are deposited in a pixel from hit to hit, causing preamplifier pulses with different rise times and therefore different recorded ToAs.^33^ As a consequence, there is a correlation between ToA and ToT. In this work the ion arrival times are calculated using the difference between two ToAs recorded from similar numbers of photons from the same energy distribution. The rise time differences are therefore effectively minimized, reducing the timewalk effect.

## Results

**Figure 2:**
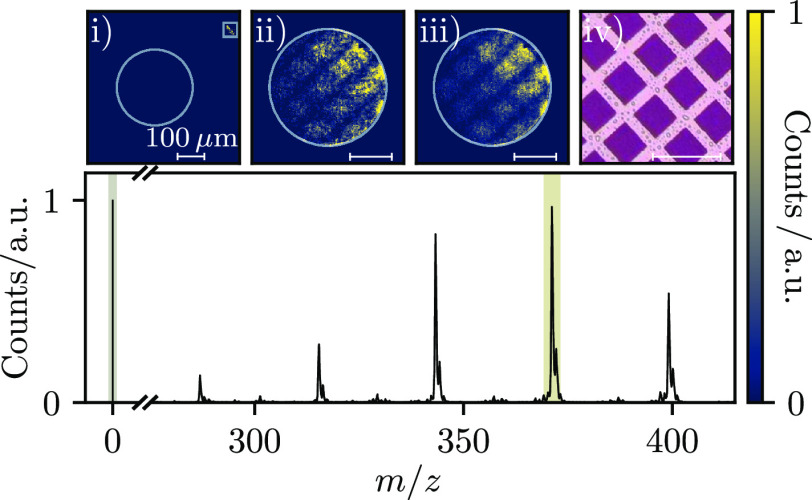
Averaged mass spectrum of rhodamine B. Integrating the Timepix3 data over ToA (shaded regions) and centroiding the pixel clusters produces images of the photon (i) and *m*/*z* signals (ii = 371.2 Da). The integrated image corresponding to all rhodamine B fragments (iii) is also shown, along with an optical microscope image of the sample (iv). The white circles in images (i–iii) represent the scintillator boundary. The white box in (i) corresponds to the area used to retrieve the trigger marker and assign the *t*_0,*n*_.

The Timepix3 camera was used to record 9000 laser desorption ionization mass spectra of rhodamine B (C_28_H_31_ClN_2_O_3_) as a continuous data set. These were sorted into individual frames by setting the light emitted by the optical fiber as a region of interest and using it to define *t*_0_ for each spectrum, as detailed in Figure 1a. Figure 2 illustrates the averaged mass spectrum. Five intense peaks are visible, which correspond to losses of Cl + CO_2_ and Cl + CO_2_ +(CH_2_)_*n*_ (*n* = 2, 4, 6, 8). The carbon isotopes of each peak are also clearly resolved. Integrating Timepix3 data over the appropriate ToA ranges allows images of the light pulse and ions to be extracted for each resolved peak (Figure 2i,ii), or over a longer window (Figure 2iii).

As flashes of light from the scintillator often illuminated more than one pixel, the recorded pixel clusters were centroided in time and space such that detected ions were assigned to single pixel coordinates. The data used for Figure 2 were clustered over 50 ToA bins and by an 8-fold nearest neighbor pixel connectivity algorithm that allowed for gaps of up to two pixels. The centroid coordinates *C* were then determined using the following equation, where *r*_*i*_ is the ToA of the *i*th pixel in a cluster:^34^
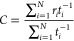
1

The above equation is a center-of-mass approach that weights each pixel in a cluster according to its ToA bin (*T*_*i*_). Since light arrives in all pixels at the same time (neglecting charge sharing effects) then the timewalk effect means that the earliest ToA’s correspond to the highest ToT’s (i.e., the brightest points of the flash). The earliest ToAs within a cluster (*T*_0_) were therefore given the most weight, and the weighting factors *t*_*i*_ were defined as
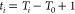
2

**Figure 3:**
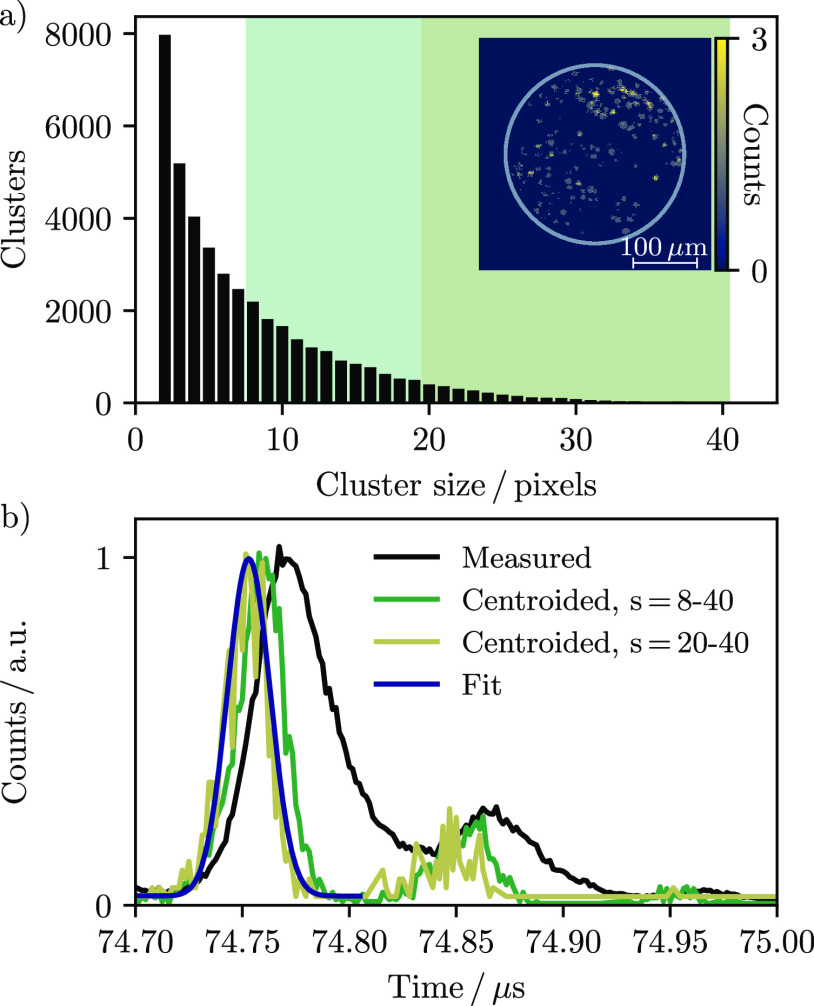
(a) Pixel cluster distribution identified from the rhodamine B signal in Figure 2 using a maximum ToA window of 50 bins (∼80 ns) and a relaxed 8-fold nearest neighbor connectivity algorithm that allows for gaps of two pixels. The inset image shows the sum of 500 laser shots and demonstrates that the brightest pixel clusters are roughly circular and contain 20 or more pixels. (b) Centroiding the measured time-of-flight spectrum (black line) over different pixel cluster size ranges *s* (green and tan lines) improves the time resolution. The sharpest resolution corresponds to the brightest pixel clusters; fitting this data to a Gaussian distribution (blue line) yields a standard deviation of 10 ns for the centroided 371.2 Da peak centered at 74.75 μs.

The ToA centroiding approach was used to mitigate the effects of the relatively small amount of visible photons per pixel area emitted by the scintillator. The mean of the ToT distribution in these experiments was only a few counts, and it was therefore easy to lose energy resolution as pixels did not always receive enough charge to go over threshold and record a hit. This is seen in the distribution of cluster sizes provided in Figure 3a, which includes many single and small pixel clusters.

The effect of centroiding on mass resolution is further demonstrated in Figure 3b, which illustrates the measured and centroided time-of-flight peaks of the 371.2 Da rhodamine B fragment. The time resolution can be further improved by counting only pixel clusters above a certain size. The inset image in Figure 3a demonstrates that the brightest pixel clusters are typically larger than 20 pixels. Centroiding and plotting only these clusters results in a Gaussian time-of-flight peak with a standard deviation of 10 ns. Applying this approach to the five principle *m*/*z* peaks in Figure 2 yields a pooled standard deviation of 9 ns and, hence, a mass resolution of 3700–4300 over the rhodamine B fragment *m*/*z* range. This value compares very well with the 9.2 ns obtained by propagating the uncertainties of the experiment, which at best include the 1.56 ns resolution of Timepix3, the 3.7 ns instrument response limited by the Exalite 404 screen, and the 8.3 ns uncertainty in assigning *t*_0_ from the trigger marker. This latter value was obtained by fitting the time-of-flight peak corresponding to the trigger flash with a Gaussian function and matches well with the 7 ns upper bound of the nominal laser pulse width. Triggering the camera with a shorter pulse, or above a particular intensity threshold, would in principle reduce this uncertainty and improve the achievable time resolution. Single photon counting experiments with Timepix3 have also been reported with a resolution of less than 2 ns, when the sensor was paired with a P47 scintillator,^35^ indicating that further enhancements are possible.

The overall resolution of 9 ns is virtually identical to the 10 ns obtained without ToT correction by Weinacht and co-workers, who used a built-in 260 ps TDC to time-stamp individual laser shots when measuring ion time-of-flight spectra.^30^ ToT correction further improved the resolution of their experiments to 5.9–8.6 ns. The trigger marking approach presented here is therefore a cost-effective substitute for an external TDC when around 10 ns resolution is required. In this case, the timing resolution of the detector was limited by the aforementioned low ToT counts. Preamplifier output current pulses were therefore shallow and introduced a several nanosecond jitter on the threshold crossing point. Increasing the photon count per pixel, for example using a more focused laser pulse, should improve this ToT information.

## Conclusion

A reflectron ion microscope incorporating a Timepix3 camera was used to measure laser desorption MSI spectra. An optical triggering system allowed ion times-of-flight recorded as a continuous data set to be referenced relative to photons split from the individual laser shots. This reduced the timewalk effect and removed the need for an external TDC, which allowed a low-cost, commercially available readout system to be used. The recorded pixel clusters represented singular ion events and were centroided using a center-of-mass approach to improve the overall time resolution to 9 ns, corresponding to a mass resolution of 4300 at 400 Da. Collectively, these results point toward the cost-effective application of multimass imaging sensors for microscope mass spectrometry imaging. The accessibility enabled by this approach directly improves the prospects of applying the benefits of ion microscopy, high throughput and mass resolution, to experiments involving congested mass spectra or time-limited samples.
